# 3-Chloro-6-[(*E*)-2-(1-phenyl­ethyl­idene)hydrazin­yl]pyridazine

**DOI:** 10.1107/S1600536810024402

**Published:** 2010-07-24

**Authors:** Abdul Qayyum Ather, M. Nawaz Tahir, Misbahul Ain Khan, Muhammad Makshoof Athar

**Affiliations:** a Department of Chemistry, Islamia University, Bahawalpur, Pakistan; b Applied Chemistry Research Center, PCSIR Laboratories Complex, Lahore 54600, Pakistan; c Department of Physics, University of Sargodha, Sargodha, Pakistan; d Institute of Chemistry, University of the Punjab, Lahore, Pakistan

## Abstract

Two independent mol­ecules are present in the asymmetric unit of the title compound, C_12_H_11_ClN_4_, (*Z*′ = 2): the dihedral angles between the phenyl and pyridizine rings are 8.35 (10) and 37.64 (6)°. In the crystal, the two mol­ecules form inversion dimers with *R*_2_^2^(8) ring motifs through inter­molecular N—H⋯N hydrogen bonds. The crystal structure is stabilized by π–π inter­actions between the pyridazine rings of symmetry-related molecules. In one of the independent mol­ecules, the centroid–centroid separations are 3.6927 (13) and 3.7961 (13) Å, whereas in the other, the separations are 3.6909 (13) and 3.9059 (13) Å.

## Related literature

For related structures, see: Ather *et al.* (2009 ▶, 2010*a* ▶,*b* ▶). For graph-set notation, see: Bernstein *et al.* (1995 ▶).
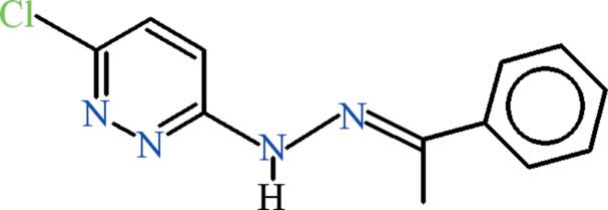


## Experimental

### 

#### Crystal data

C_12_H_11_ClN_4_
*M* *_r_* = 246.70
Monoclinic, 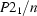

*a* = 12.8006 (6) Å
*b* = 7.4703 (5) Å
*c* = 24.9520 (14) Å
β = 90.737 (2)°
*V* = 2385.8 (2) Å^3^
*Z* = 8
Mo *K*α radiation
μ = 0.30 mm^−1^
*T* = 296 K
0.30 × 0.14 × 0.14 mm

#### Data collection

Bruker Kappa APEXII CCD diffractometer
Absorption correction: multi-scan (*SADABS*; Bruker, 2009 ▶) *T*_min_ = 0.982, *T*_max_ = 0.988
22389 measured reflections
5900 independent reflections
3179 reflections with *I* > 2σ(*I*)
*R*_int_ = 0.054

#### Refinement

*R*[*F* ^2^ > 2σ(*F* ^2^)] = 0.049
*wR*(*F* ^2^) = 0.148
*S* = 1.06
5900 reflections
309 parameters
H-atom parameters constrained
Δρ_max_ = 0.22 e Å^−3^
Δρ_min_ = −0.20 e Å^−3^

### 

Data collection: *APEX2* (Bruker, 2009 ▶); cell refinement: *SAINT* (Bruker, 2009 ▶); data reduction: *SAINT*; program(s) used to solve structure: *SHELXS97* (Sheldrick, 2008 ▶); program(s) used to refine structure: *SHELXL97* (Sheldrick, 2008 ▶); molecular graphics: *ORTEP-3 for Windows* (Farrugia, 1997 ▶) and *PLATON* (Spek, 2009 ▶); software used to prepare material for publication: *WinGX* publication routines (Farrugia, 1999 ▶) and *PLATON*.

## Supplementary Material

Crystal structure: contains datablocks global, I. DOI: 10.1107/S1600536810024402/si2272sup1.cif

Structure factors: contains datablocks I. DOI: 10.1107/S1600536810024402/si2272Isup2.hkl

Additional supplementary materials:  crystallographic information; 3D view; checkCIF report

## Figures and Tables

**Table 1:** Hydrogen-bond geometry (Å, °)

*D*—H⋯*A*	*D*—H	H⋯*A*	*D*⋯*A*	*D*—H⋯*A*
N2—H2*A*⋯N7^i^	0.86	2.35	3.102 (2)	146
N6—H6*A*⋯N3^i^	0.86	2.35	3.138 (2)	153

Symmetry code: (i) 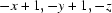.

**Fig. 1.**
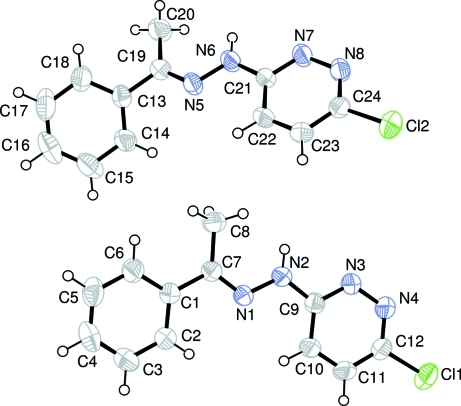
View of the title compound with the atom numbering scheme. The thermal ellipsoids are drawn at the 50% probability level. H-atoms are shown as small spheres of arbitrary radii.

**Fig. 2.**
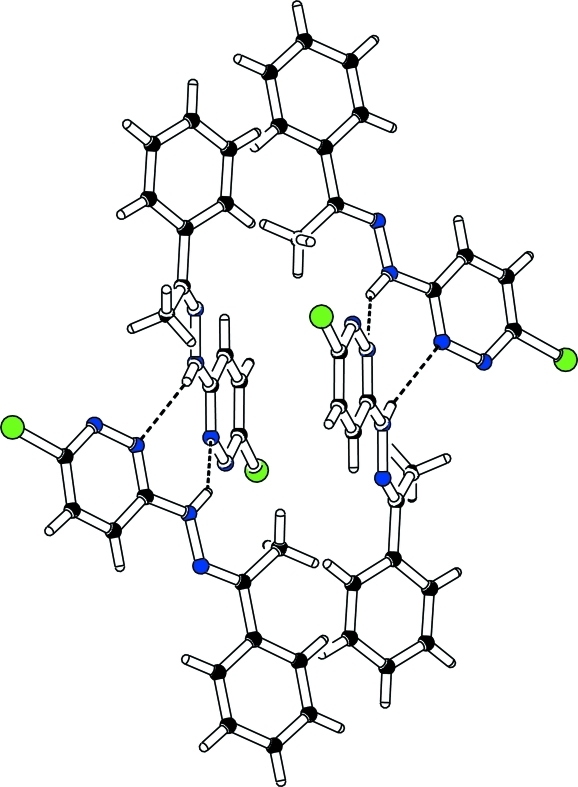
Packing diagram of the title compound (PLATON: Spek, 2009) showing that the molecules form dimers.

## supplementary crystallographic information

### Comment

In continuation to pyridazine derivatives (Ather *et al.*, 2009, 2010*a*, 2010*b*), the title compound (I, Fig. 1) is being reported here.

The title compound consists of two molecules in the crystallographic asymmetric unit which differ from each other geometrically. In one molecule, the phenyl ring A (C1—C6) of 1-phenylethanimine, the central part B (C8/C7/N1/N2) and the chloro substituated pyridazine C (C9–C12/N3/N4/CL1) are planar with r. m. s. deviation of 0.0063, 0.0009 and 0.0053 Å respectively. The dihedral angle between A/B, A/C and B/C is 4.66 (13)°, 8.40 (9)° and 4.39 (10)°, respectively. In second molecule, the phenyl ring D (C13—C18) of 1-phenylethanimine, the central part E (C20/C19/N5/N6) and the chloro substituated pyridazine F (C21–C24/N7/N8/CL2) are planar with r. m. s. deviation of 0.0056, 0.0009 and 0.0033 Å respectively. The dihedral angle between D/E, D/F and E/F is 38.46 (7)°, 37.77 (5)° and 0.75 (9)°, respectively. These values confirm that the selection of crystal system and space group is correct. The two molecules form dimers (Fig. 2) with each other through intermolecular H-bondings of N—H···N type with *R*_2_^2^(8) ring motifs (Bernstein *et al.*, 1995). There exist π–π interactions between the similar pyridazine rings. In first molecule the distance between the centroids of pyridazine rings is 3.6927 (13) and 3.7961 (13) Å, whereas in the second molecules the separation between the pyridazine rings is 3.6909 (13) and 3.9059 (13) Å.

### Experimental

3-Chloro-6-hydrazinylpyridazine (0.5 g, 3.46 mmol) was dissolved in ethanol (15 ml). 1-Phenylethanone (0.416 g, 3.46 mmol) was added to the solution and refluxed for 2 h. The reaction was monitored by TLC. After the completion, the reaction mixture was concentrated under vacuum. Distilled water (20 ml) was added to the resulting concentrated mixture, which give rise to precipitates. The filtered precipitate were dried and re-crystallized in ethanol to obtaine the light yellow needles of title compound (I).

### Refinement

The H-atoms were positioned geometrically with N–H = 0.86 Å, C–H = 0.93 and C–H = 0.96 Å for aromatic rings and methyl atoms and constrained to ride on their parent atoms with *U*_iso_(H) = x*U*_eq_(C, N), where x = 1.5 for methyl and x = 1.2 for all other H-atoms.

### Crystal data

**Table:**

C_12_H_11_ClN_4_	*F*(000) = 1024
*M**_r_* = 246.70	*D*_x_ = 1.374 Mg m^−^^3^
Monoclinic, *P*2_1_/*n*	Mo *K*α radiation, λ = 0.71073 Å
Hall symbol: -P 2yn	Cell parameters from 3179 reflections
*a* = 12.8006 (6) Å	θ = 2.9–28.3°
*b* = 7.4703 (5) Å	µ = 0.30 mm^−^^1^
*c* = 24.9520 (14) Å	*T* = 296 K
β = 90.737 (2)°	Needle, light yellow
*V* = 2385.8 (2) Å^3^	0.30 × 0.14 × 0.14 mm
*Z* = 8	

### Data collection

**Table:**

Bruker KAPPA APEXII CCD diffractometer	5900 independent reflections
Radiation source: fine-focus sealed tube	3179 reflections with *I* > 2σ(*I*)
graphite	*R*_int_ = 0.054
Detector resolution: 7.40 pixels mm^-1^	θ_max_ = 28.3°, θ_min_ = 2.9°
ω scans	*h* = −17→16
Absorption correction: multi-scan (*SADABS*; Bruker, 2009)	*k* = −9→9
*T*_min_ = 0.982, *T*_max_ = 0.988	*l* = −33→33
22389 measured reflections	

### Refinement

**Table:**

Refinement on *F*^2^	Primary atom site location: structure-invariant direct methods
Least-squares matrix: full	Secondary atom site location: difference Fourier map
*R*[*F*^2^ > 2σ(*F*^2^)] = 0.049	Hydrogen site location: inferred from neighbouring sites
*wR*(*F*^2^) = 0.148	H-atom parameters constrained
*S* = 1.06	*w* = 1/[σ^2^(*F*_o_^2^) + (0.0601*P*)^2^ + 0.068*P*] where *P* = (*F*_o_^2^ + 2*F*_c_^2^)/3
5900 reflections	(Δ/σ)_max_ = 0.001
309 parameters	Δρ_max_ = 0.22 e Å^−^^3^
0 restraints	Δρ_min_ = −0.19 e Å^−^^3^

### Special details

**Table:**

Geometry. Bond distances, angles *etc*. have been calculated using the rounded fractional coordinates. All su's are estimated from the variances of the (full) variance-covariance matrix. The cell e.s.d.'s are taken into account in the estimation of distances, angles and torsion angles
Refinement. Refinement of *F*^2^ against ALL reflections. The weighted *R*-factor *wR* and goodness of fit *S* are based on *F*^2^, conventional *R*-factors *R* are based on *F*, with *F* set to zero for negative *F*^2^. The threshold expression of *F*^2^ > σ(*F*^2^) is used only for calculating *R*-factors(gt) *etc*. and is not relevant to the choice of reflections for refinement. *R*-factors based on *F*^2^ are statistically about twice as large as those based on *F*, and *R*- factors based on ALL data will be even larger.

### Fractional atomic coordinates and isotropic or equivalent isotropic displacement parameters (Å^2^)

**Table:**

	*x*	*y*	*z*	*U*_iso_*/*U*_eq_	
Cl1	1.14191 (4)	0.12613 (9)	−0.08803 (2)	0.0681 (3)	
N1	0.87290 (12)	0.3777 (2)	0.12027 (7)	0.0439 (6)	
N2	0.84605 (12)	0.3627 (3)	0.06698 (7)	0.0487 (6)	
N3	0.89323 (13)	0.2970 (3)	−0.01854 (7)	0.0510 (7)	
N4	0.96364 (13)	0.2410 (3)	−0.05514 (7)	0.0521 (7)	
C1	0.83894 (15)	0.4538 (3)	0.20972 (8)	0.0409 (7)	
C2	0.93426 (16)	0.3832 (3)	0.22711 (9)	0.0543 (9)	
C3	0.96554 (18)	0.3954 (4)	0.27995 (10)	0.0660 (10)	
C4	0.9016 (2)	0.4749 (3)	0.31703 (9)	0.0654 (10)	
C5	0.8075 (2)	0.5411 (3)	0.30105 (10)	0.0662 (10)	
C6	0.77589 (17)	0.5314 (3)	0.24801 (9)	0.0561 (8)	
C7	0.80578 (14)	0.4423 (3)	0.15264 (8)	0.0412 (7)	
C8	0.69860 (15)	0.5040 (3)	0.13580 (9)	0.0578 (9)	
C9	0.92084 (15)	0.3016 (3)	0.03289 (8)	0.0424 (7)	
C10	1.01991 (15)	0.2491 (3)	0.05149 (9)	0.0510 (8)	
C11	1.08926 (16)	0.1954 (3)	0.01446 (9)	0.0531 (8)	
C12	1.05685 (16)	0.1951 (3)	−0.03850 (9)	0.0476 (8)	
Cl2	0.62276 (4)	0.87288 (9)	−0.08693 (2)	0.0659 (3)	
N5	0.36036 (13)	0.5987 (2)	0.12034 (7)	0.0470 (6)	
N6	0.32914 (13)	0.6283 (3)	0.06798 (7)	0.0498 (6)	
N7	0.37228 (12)	0.7131 (3)	−0.01655 (7)	0.0496 (6)	
N8	0.44215 (13)	0.7711 (3)	−0.05326 (7)	0.0504 (7)	
C13	0.33286 (15)	0.5047 (3)	0.20856 (8)	0.0457 (7)	
C14	0.43119 (16)	0.4306 (3)	0.21617 (9)	0.0590 (9)	
C15	0.4697 (2)	0.3949 (4)	0.26709 (11)	0.0737 (10)	
C16	0.4102 (3)	0.4353 (4)	0.31069 (11)	0.0824 (11)	
C17	0.3130 (3)	0.5056 (4)	0.30392 (11)	0.0847 (11)	
C18	0.27360 (19)	0.5411 (4)	0.25298 (10)	0.0659 (10)	
C19	0.29251 (15)	0.5417 (3)	0.15356 (8)	0.0447 (7)	
C20	0.18083 (15)	0.5048 (4)	0.14036 (9)	0.0652 (10)	
C21	0.40365 (15)	0.6868 (3)	0.03380 (8)	0.0413 (7)	
C22	0.50712 (15)	0.7153 (3)	0.05069 (9)	0.0504 (8)	
C23	0.57536 (16)	0.7719 (3)	0.01398 (9)	0.0544 (9)	
C24	0.53849 (15)	0.7979 (3)	−0.03786 (8)	0.0452 (8)	
H2	0.97743	0.32685	0.20260	0.0652*	
H2A	0.78456	0.39078	0.05550	0.0584*	
H3	1.03011	0.34981	0.29067	0.0790*	
H4	0.92279	0.48315	0.35274	0.0784*	
H5	0.76379	0.59361	0.32608	0.0794*	
H6	0.71119	0.57763	0.23780	0.0673*	
H8A	0.65139	0.40417	0.13589	0.0867*	
H8B	0.70128	0.55384	0.10038	0.0867*	
H8C	0.67465	0.59352	0.16036	0.0867*	
H10	1.03718	0.25111	0.08780	0.0612*	
H11	1.15646	0.15993	0.02430	0.0637*	
H6A	0.26572	0.61074	0.05748	0.0597*	
H14	0.47190	0.40447	0.18658	0.0708*	
H15	0.53550	0.34397	0.27169	0.0884*	
H16	0.43643	0.41443	0.34507	0.0988*	
H17	0.27255	0.53020	0.33372	0.1014*	
H18	0.20706	0.58948	0.24880	0.0790*	
H20A	0.17625	0.43717	0.10771	0.0976*	
H20B	0.14407	0.61598	0.13594	0.0976*	
H20C	0.15016	0.43759	0.16892	0.0976*	
H22	0.52778	0.69562	0.08607	0.0605*	
H23	0.64498	0.79301	0.02305	0.0652*	

### Atomic displacement parameters (Å^2^)

**Table:**

	*U*^11^	*U*^22^	*U*^33^	*U*^12^	*U*^13^	*U*^23^
Cl1	0.0605 (4)	0.0800 (5)	0.0644 (4)	0.0011 (3)	0.0209 (3)	−0.0083 (3)
N1	0.0420 (9)	0.0523 (11)	0.0373 (10)	0.0006 (8)	−0.0031 (8)	−0.0047 (9)
N2	0.0408 (10)	0.0648 (13)	0.0403 (10)	0.0052 (9)	−0.0023 (8)	−0.0052 (9)
N3	0.0482 (10)	0.0663 (13)	0.0386 (11)	0.0077 (9)	0.0006 (8)	−0.0020 (9)
N4	0.0502 (11)	0.0642 (13)	0.0420 (11)	0.0021 (9)	0.0035 (9)	−0.0037 (9)
C1	0.0425 (11)	0.0386 (12)	0.0417 (12)	−0.0036 (9)	0.0013 (9)	−0.0010 (10)
C2	0.0477 (13)	0.0680 (17)	0.0472 (14)	0.0022 (11)	−0.0001 (10)	−0.0013 (12)
C3	0.0593 (15)	0.082 (2)	0.0562 (16)	0.0015 (13)	−0.0146 (12)	0.0046 (14)
C4	0.0936 (19)	0.0618 (17)	0.0404 (14)	−0.0081 (15)	−0.0097 (13)	−0.0022 (12)
C5	0.0921 (19)	0.0604 (17)	0.0462 (15)	0.0132 (14)	0.0105 (13)	−0.0080 (13)
C6	0.0621 (14)	0.0584 (16)	0.0479 (14)	0.0130 (12)	0.0018 (11)	−0.0064 (12)
C7	0.0406 (11)	0.0403 (12)	0.0426 (12)	−0.0034 (9)	−0.0008 (9)	−0.0028 (10)
C8	0.0454 (12)	0.0738 (18)	0.0540 (15)	0.0110 (12)	−0.0040 (10)	−0.0104 (13)
C9	0.0422 (12)	0.0443 (13)	0.0405 (12)	−0.0052 (9)	−0.0011 (9)	−0.0030 (10)
C10	0.0395 (12)	0.0689 (16)	0.0445 (13)	−0.0028 (11)	−0.0064 (10)	−0.0072 (11)
C11	0.0350 (11)	0.0679 (17)	0.0563 (15)	−0.0024 (11)	−0.0041 (10)	−0.0040 (12)
C12	0.0445 (12)	0.0505 (14)	0.0479 (14)	−0.0034 (10)	0.0071 (10)	−0.0031 (11)
Cl2	0.0597 (4)	0.0801 (5)	0.0584 (4)	−0.0044 (3)	0.0162 (3)	0.0076 (3)
N5	0.0486 (10)	0.0556 (12)	0.0366 (10)	−0.0048 (8)	−0.0034 (8)	0.0064 (9)
N6	0.0419 (10)	0.0677 (13)	0.0395 (10)	−0.0068 (9)	−0.0062 (8)	0.0074 (9)
N7	0.0458 (10)	0.0656 (13)	0.0374 (10)	−0.0068 (9)	−0.0032 (8)	0.0041 (9)
N8	0.0507 (11)	0.0614 (13)	0.0390 (10)	−0.0037 (9)	0.0006 (8)	0.0047 (9)
C13	0.0501 (12)	0.0482 (14)	0.0387 (12)	−0.0074 (10)	0.0005 (10)	0.0024 (10)
C14	0.0483 (13)	0.0776 (19)	0.0511 (15)	−0.0038 (12)	−0.0009 (10)	0.0149 (13)
C15	0.0681 (16)	0.089 (2)	0.0636 (18)	−0.0096 (14)	−0.0178 (14)	0.0238 (16)
C16	0.114 (2)	0.086 (2)	0.0465 (17)	−0.0165 (19)	−0.0203 (16)	0.0158 (15)
C17	0.118 (2)	0.093 (2)	0.0435 (17)	0.009 (2)	0.0174 (16)	−0.0029 (15)
C18	0.0769 (17)	0.0728 (19)	0.0482 (15)	0.0164 (14)	0.0107 (12)	0.0031 (13)
C19	0.0458 (12)	0.0435 (13)	0.0447 (13)	−0.0009 (10)	−0.0011 (10)	0.0003 (10)
C20	0.0483 (13)	0.088 (2)	0.0591 (16)	−0.0086 (13)	−0.0044 (11)	0.0115 (14)
C21	0.0414 (11)	0.0439 (13)	0.0384 (12)	0.0027 (9)	−0.0038 (9)	0.0023 (10)
C22	0.0409 (12)	0.0673 (16)	0.0427 (13)	−0.0010 (11)	−0.0090 (10)	0.0045 (11)
C23	0.0372 (12)	0.0737 (18)	0.0520 (15)	0.0000 (11)	−0.0047 (10)	0.0025 (12)
C24	0.0446 (12)	0.0464 (14)	0.0446 (13)	0.0014 (10)	0.0034 (10)	0.0006 (10)

### Geometric parameters (Å, °)

**Table:**

Cl1—C12	1.736 (2)	C4—H4	0.9300
Cl2—C24	1.735 (2)	C5—H5	0.9300
N1—C7	1.281 (3)	C6—H6	0.9300
N1—N2	1.374 (2)	C8—H8A	0.9600
N2—C9	1.367 (3)	C8—H8B	0.9600
N3—C9	1.327 (3)	C8—H8C	0.9600
N3—N4	1.357 (2)	C10—H10	0.9300
N4—C12	1.304 (3)	C11—H11	0.9300
N2—H2A	0.8600	C13—C14	1.386 (3)
N5—C19	1.281 (3)	C13—C19	1.486 (3)
N5—N6	1.379 (2)	C13—C18	1.378 (3)
N6—C21	1.360 (3)	C14—C15	1.383 (4)
N7—C21	1.329 (3)	C15—C16	1.370 (4)
N7—N8	1.359 (2)	C16—C17	1.359 (5)
N8—C24	1.302 (3)	C17—C18	1.387 (4)
N6—H6A	0.8600	C19—C20	1.489 (3)
C1—C6	1.386 (3)	C21—C22	1.401 (3)
C1—C2	1.393 (3)	C22—C23	1.342 (3)
C1—C7	1.483 (3)	C23—C24	1.385 (3)
C2—C3	1.376 (3)	C14—H14	0.9300
C3—C4	1.378 (3)	C15—H15	0.9300
C4—C5	1.357 (4)	C16—H16	0.9300
C5—C6	1.381 (3)	C17—H17	0.9300
C7—C8	1.502 (3)	C18—H18	0.9300
C9—C10	1.401 (3)	C20—H20A	0.9600
C10—C11	1.351 (3)	C20—H20B	0.9600
C11—C12	1.380 (3)	C20—H20C	0.9600
C2—H2	0.9300	C22—H22	0.9300
C3—H3	0.9300	C23—H23	0.9300
			
Cl1···C8^i^	3.645 (2)	C16···H20B^xii^	2.8200
Cl1···C9^ii^	3.574 (2)	C18···H20C	2.7200
Cl1···C10^ii^	3.609 (2)	C20···H18	2.7900
Cl2···C22^iii^	3.617 (2)	C20···H10^xi^	2.9400
Cl2···C21^iii^	3.564 (2)	C20···H6A	2.4800
Cl1···H5^iv^	3.1300	C22···H8B	3.0100
Cl1···H8B^i^	3.1400	H2···H20C^x^	2.5200
Cl1···H16^iv^	3.1100	H2···N1	2.4700
Cl2···H4^v^	3.1400	H2A···H8B	1.9800
Cl2···H17^vi^	2.8700	H2A···C8	2.4500
N2···N7^vii^	3.102 (2)	H2A···N7^vii^	2.3500
N3···N6^vii^	3.138 (2)	H4···Cl2^xiii^	3.1400
N4···C20^vii^	3.383 (3)	H5···C7^viii^	2.8100
N6···N3^vii^	3.138 (2)	H5···Cl1^xiv^	3.1300
N7···N2^vii^	3.102 (2)	H6···H8C	1.9900
N8···C8^vii^	3.408 (3)	H6···C2^viii^	3.0800
N1···H2	2.4700	H6···C8	2.6100
N1···H10	2.4500	H6A···C20	2.4800
N2···H8B	2.4900	H6A···H20B	2.5200
N3···H6A^vii^	2.3500	H6A···N3^vii^	2.3500
N4···H16^iv^	2.7700	H6A···H20A	2.1400
N4···H20B^vii^	2.6500	H8A···N8^vii^	2.7100
N5···H22	2.4300	H8B···C22	3.0100
N5···H14	2.6100	H8B···N2	2.4900
N6···H20A	2.6300	H8B···Cl1^i^	3.1400
N6···H20B	2.9300	H8B···H2A	1.9800
N7···H2A^vii^	2.3500	H8B···H22	2.4800
N8···H8A^vii^	2.7100	H8C···C4^viii^	3.0700
C5···C7^viii^	3.530 (3)	H8C···C6	2.5700
C7···C5^ix^	3.530 (3)	H8C···H6	1.9900
C8···N8^vii^	3.408 (3)	H10···H20A^x^	2.3100
C8···C22	3.588 (3)	H10···N1	2.4500
C8···Cl1^i^	3.645 (2)	H10···C20^x^	2.9400
C9···Cl1^ii^	3.574 (2)	H14···N5	2.6100
C10···C20^x^	3.563 (3)	H16···H20B^xii^	2.5000
C10···C12^ii^	3.475 (3)	H16···Cl1^xiv^	3.1100
C10···Cl1^ii^	3.609 (2)	H16···N4^xiv^	2.7700
C11···C12^ii^	3.521 (3)	H17···Cl2^xv^	2.8700
C12···C11^ii^	3.521 (3)	H18···C20	2.7900
C12···C10^ii^	3.475 (3)	H18···H20C	2.4000
C20···N4^vii^	3.383 (3)	H20A···N6	2.6300
C20···C10^xi^	3.563 (3)	H20A···C10^xi^	2.8100
C21···Cl2^iii^	3.564 (2)	H20A···H6A	2.1400
C22···Cl2^iii^	3.617 (2)	H20A···H10^xi^	2.3100
C22···C8	3.588 (3)	H20B···N6	2.9300
C23···C24^iii^	3.582 (3)	H20B···H6A	2.5200
C24···C23^iii^	3.582 (3)	H20B···C16^xvi^	2.8200
C2···H6^ix^	3.0800	H20B···H16^xvi^	2.5000
C4···H8C^ix^	3.0700	H20B···N4^vii^	2.6500
C6···H8C	2.5700	H20C···C18	2.7200
C7···H5^ix^	2.8100	H20C···H2^xi^	2.5200
C8···H2A	2.4500	H20C···H18	2.4000
C8···H22	2.8800	H22···N5	2.4300
C8···H6	2.6100	H22···C8	2.8800
C10···H20A^x^	2.8100	H22···H8B	2.4800
			
N2—N1—C7	118.65 (16)	H8A—C8—H8B	109.00
N1—N2—C9	117.45 (16)	C7—C8—H8C	109.00
N4—N3—C9	119.19 (17)	C11—C10—H10	121.00
N3—N4—C12	118.65 (18)	C9—C10—H10	121.00
N1—N2—H2A	121.00	C10—C11—H11	121.00
C9—N2—H2A	121.00	C12—C11—H11	121.00
N6—N5—C19	118.40 (17)	C14—C13—C18	118.5 (2)
N5—N6—C21	116.66 (16)	C14—C13—C19	120.34 (18)
N8—N7—C21	119.40 (16)	C18—C13—C19	121.17 (19)
N7—N8—C24	118.61 (17)	C13—C14—C15	121.0 (2)
N5—N6—H6A	122.00	C14—C15—C16	119.5 (3)
C21—N6—H6A	122.00	C15—C16—C17	120.3 (3)
C6—C1—C7	121.62 (18)	C16—C17—C18	120.6 (3)
C2—C1—C6	117.33 (19)	C13—C18—C17	120.1 (2)
C2—C1—C7	121.04 (18)	N5—C19—C13	115.40 (17)
C1—C2—C3	121.1 (2)	N5—C19—C20	125.14 (19)
C2—C3—C4	120.3 (2)	C13—C19—C20	119.42 (18)
C3—C4—C5	119.5 (2)	N6—C21—N7	115.77 (17)
C4—C5—C6	120.7 (2)	N7—C21—C22	122.35 (19)
C1—C6—C5	121.1 (2)	N6—C21—C22	121.88 (19)
N1—C7—C1	116.18 (17)	C21—C22—C23	117.6 (2)
C1—C7—C8	119.99 (17)	C22—C23—C24	117.69 (19)
N1—C7—C8	123.83 (18)	N8—C24—C23	124.33 (19)
N2—C9—N3	115.49 (17)	Cl2—C24—N8	115.87 (15)
N3—C9—C10	122.81 (19)	Cl2—C24—C23	119.79 (15)
N2—C9—C10	121.70 (18)	C13—C14—H14	119.00
C9—C10—C11	117.2 (2)	C15—C14—H14	119.00
C10—C11—C12	117.61 (19)	C14—C15—H15	120.00
Cl1—C12—C11	119.91 (16)	C16—C15—H15	120.00
Cl1—C12—N4	115.54 (17)	C15—C16—H16	120.00
N4—C12—C11	124.5 (2)	C17—C16—H16	120.00
C3—C2—H2	119.00	C16—C17—H17	120.00
C1—C2—H2	119.00	C18—C17—H17	120.00
C2—C3—H3	120.00	C13—C18—H18	120.00
C4—C3—H3	120.00	C17—C18—H18	120.00
C3—C4—H4	120.00	C19—C20—H20A	109.00
C5—C4—H4	120.00	C19—C20—H20B	109.00
C6—C5—H5	120.00	C19—C20—H20C	109.00
C4—C5—H5	120.00	H20A—C20—H20B	109.00
C1—C6—H6	119.00	H20A—C20—H20C	109.00
C5—C6—H6	119.00	H20B—C20—H20C	109.00
C7—C8—H8A	109.00	C21—C22—H22	121.00
C7—C8—H8B	109.00	C23—C22—H22	121.00
H8A—C8—H8C	109.00	C22—C23—H23	121.00
H8B—C8—H8C	109.00	C24—C23—H23	121.00
			
C7—N1—N2—C9	176.9 (2)	C7—C1—C6—C5	179.9 (2)
N2—N1—C7—C1	179.52 (18)	C1—C2—C3—C4	1.4 (4)
N2—N1—C7—C8	−0.3 (3)	C2—C3—C4—C5	0.0 (4)
N1—N2—C9—N3	−177.00 (19)	C3—C4—C5—C6	−0.8 (4)
N1—N2—C9—C10	2.5 (3)	C4—C5—C6—C1	0.1 (4)
C9—N3—N4—C12	−0.7 (3)	N3—C9—C10—C11	1.3 (3)
N4—N3—C9—N2	178.8 (2)	N2—C9—C10—C11	−178.2 (2)
N4—N3—C9—C10	−0.7 (3)	C9—C10—C11—C12	−0.5 (3)
N3—N4—C12—Cl1	−179.75 (17)	C10—C11—C12—Cl1	−179.57 (17)
N3—N4—C12—C11	1.5 (4)	C10—C11—C12—N4	−0.9 (4)
N6—N5—C19—C13	177.23 (18)	C18—C13—C14—C15	−0.5 (4)
N6—N5—C19—C20	−0.3 (3)	C19—C13—C14—C15	−179.8 (2)
C19—N5—N6—C21	−179.3 (2)	C14—C13—C18—C17	0.7 (4)
N5—N6—C21—C22	0.2 (3)	C19—C13—C18—C17	−179.9 (3)
N5—N6—C21—N7	179.46 (19)	C14—C13—C19—N5	−37.7 (3)
N8—N7—C21—N6	−179.8 (2)	C14—C13—C19—C20	140.0 (2)
C21—N7—N8—C24	0.5 (3)	C18—C13—C19—N5	143.0 (2)
N8—N7—C21—C22	−0.5 (3)	C18—C13—C19—C20	−39.3 (3)
N7—N8—C24—Cl2	−179.70 (17)	C13—C14—C15—C16	−0.8 (4)
N7—N8—C24—C23	−0.3 (4)	C14—C15—C16—C17	1.7 (5)
C6—C1—C2—C3	−2.0 (3)	C15—C16—C17—C18	−1.4 (5)
C7—C1—C2—C3	179.3 (2)	C16—C17—C18—C13	0.2 (5)
C2—C1—C7—N1	−5.4 (3)	N6—C21—C22—C23	179.5 (2)
C2—C1—C7—C8	174.3 (2)	N7—C21—C22—C23	0.3 (3)
C6—C1—C7—N1	175.9 (2)	C21—C22—C23—C24	0.0 (3)
C6—C1—C7—C8	−4.3 (3)	C22—C23—C24—Cl2	179.42 (18)
C2—C1—C6—C5	1.2 (3)	C22—C23—C24—N8	0.0 (4)

Symmetry codes: (i) −*x*+2, −*y*+1, −*z*; (ii) −*x*+2, −*y*, −*z*; (iii) −*x*+1, −*y*+2, −*z*; (iv) *x*+1/2, −*y*+1/2, *z*−1/2; (v) *x*−1/2, −*y*+3/2, *z*−1/2; (vi) *x*+1/2, −*y*+3/2, *z*−1/2; (vii) −*x*+1, −*y*+1, −*z*; (viii) −*x*+3/2, *y*+1/2, −*z*+1/2; (ix) −*x*+3/2, *y*−1/2, −*z*+1/2; (x) *x*+1, *y*, *z*; (xi) *x*−1, *y*, *z*; (xii) −*x*+1/2, *y*−1/2, −*z*+1/2; (xiii) *x*+1/2, −*y*+3/2, *z*+1/2; (xiv) *x*−1/2, −*y*+1/2, *z*+1/2; (xv) *x*−1/2, −*y*+3/2, *z*+1/2; (xvi) −*x*+1/2, *y*+1/2, −*z*+1/2.

### Hydrogen-bond geometry (Å, °)

**Table:**

*D*—H···*A*	*D*—H	H···*A*	*D*···*A*	*D*—H···*A*
N2—H2A···N7^vii^	0.8600	2.3500	3.102 (2)	146.00
N6—H6A···N3^vii^	0.8600	2.3500	3.138 (2)	153.00

Symmetry codes: (vii) −*x*+1, −*y*+1, −*z*.
